# Prediction of Neurologically Intact Survival in Cardiac Arrest Patients without Pre-Hospital Return of Spontaneous Circulation: Machine Learning Approach

**DOI:** 10.3390/jcm10051089

**Published:** 2021-03-05

**Authors:** Dong-Woo Seo, Hahn Yi, Hyun-Jin Bae, Youn-Jung Kim, Chang-Hwan Sohn, Shin Ahn, Kyoung-Soo Lim, Namkug Kim, Won-Young Kim

**Affiliations:** 1 Asan Medical Center, Department of Emergency Medicine, College of Medicine, University of Ulsan, Seoul 05505, Korea; leiseo@gmail.com (D.-W.S.); dbswjdsla@gmail.com (Y.-J.K.); schwan97@gmail.com (C.-H.S.); ans1023@gmail.com (S.A.); kslim@amc.seoul.kr (K.-S.L.); 2 Asan Medical Center, Department of Information Medicine, College of Medicine, University of Ulsan, Seoul 05505, Korea; 3 Asan Medical Center, Asan Institute for Life Sciences, Seoul 05505, Korea; hahn.yi@gmail.com; 4 Asan Medical Center, Department of Medicine, College of Medicine, University of Ulsan, Seoul 05505, Korea; hjbae.astro@gmail.com; 5 Asan Medical Center, Department of Convergence Medicine, College of Medicine, University of Ulsan, Seoul 05505, Korea

**Keywords:** emergency departments, machine learning, out-of-hospital cardiac arrest, outcomes, resuscitation, targeted temperature management

## Abstract

Current multimodal approaches for the prognostication of out-of-hospital cardiac arrest (OHCA) are based mainly on the prediction of poor neurological outcomes; however, it is challenging to identify patients expected to have a favorable outcome, especially before the return of spontaneous circulation (ROSC). We developed and validated a machine learning-based system to predict good outcome in OHCA patients before ROSC. This prospective, multicenter, registry-based study analyzed non-traumatic OHCA data collected between October 2015 and June 2017. We used information available before ROSC as predictor variables, and the primary outcome was neurologically intact survival at discharge, defined as cerebral performance category 1 or 2. The developed models’ robustness were evaluated and compared with various score metrics to confirm their performance. The model using a voting classifier had the best performance in predicting good neurological outcome (area under the curve = 0.926). We confirmed that the six top-weighted variables predicting neurological outcomes, such as several duration variables after the instant of OHCA and several electrocardiogram variables in the voting classifier model, showed significant differences between the two neurological outcome groups. These findings demonstrate the potential utility of a machine learning model to predict good neurological outcome of OHCA patients before ROSC.

## 1. Introduction

Despite advances in cardiac arrest resuscitation, the rate of survival to discharge in patients with out-of-hospital cardiac arrest (OHCA) who receive cardiopulmonary resuscitation (CPR) remains low, ranging from 6.7% to 10.8%, and only 5% of survivors experience full neurological recovery [1,2,3,4,5].

Current guidelines recommend that neurological outcomes in patients with OHCA should be predicted based on multimodal approaches to minimize the risk of falsely predicting poor outcomes, which may lead to the withdrawal of life-sustaining treatment [6]. Multimodal approaches include the use of clinical examination, serum biomarkers, electrophysiological tests, and neuroimaging. However, one of the most pressing issues for relatives and healthcare workers is to rapidly obtain reliable information regarding the probability of achieving favorable neurological outcomes [7]. Although numerous studies have focused on developing prediction models for poor outcomes [8,9], it is also essential to develop strategies for predicting favorable neurological outcomes to appropriately tailor medical therapies for individual patients. Furthermore, extracorporeal membrane oxygenation (ECMO)-CPR in the emergency departments (EDs) has been suggested as a potential rescue therapy in patients with refractory OHCA and suspected cardiogenic etiology of the arrest. Before the return of spontaneous circulation (ROSC), reliable information for expected good neurological survival can impact the choices of appropriate care provided by caregivers and the provision of advanced interventions by physicians. The OHCA risk score using variables available at hospital admission showed an area under the curve (AUC) of 0.88. However, this score was based on a small number of patients (*n* = 130) who were relatively young compared to those in other studies [10]. The Cardiac Arrest Hospital Prognosis (CAHP) score, which was developed using a large number of patients, performs similarly (AUC 0.93) [11]. Unlike our study, OHCA and CAHP scores include a patient population that has already achieved ROSC [1,11]. The recently published shockable rhythm-witness-age-pH (SWAP) score, based on a large cohort, also showed a similar diagnostic capability (when SWAP score > 2, sensitivity = 75.0% and specificity = 89.5% for poor neurological outcome) [1].

Newer computational methods, namely machine learning (ML), may allow more accurate prediction than risk assessment tools developed using standard methods. Targeted machine learning (ML) algorithms triggered by patient data have been increasingly developed as clinical decision support tools in various diseases including sepsis, gastrointestinal bleeding, and acute kidney injury [12,13,14,15,16,17,18,19]. Although several ML models for predicting the development of in-hospital cardiac arrest have been reported in the resuscitation field, there remains a paucity of data regarding an ML system for the prediction of good outcomes in patients with OHCA. Especially in cardiac arrest patients without pre-hospital ROSC, the probability of achieving favorable neurological outcomes is an important issue. However, the few recent studies of deep-learning-based prognostic systems did not exclude patients with pre-hospital ROSC [20].

Given the complexity and time dependency of OHCA patients receiving CPR, ML-based methods are expected to provide a good foundation for developing tools for the prediction of favorable outcomes. The objective of this study was to develop and validate an ML-based prognostic model for good neurological outcome in patients with OHCA before ROSC using a nationwide multicenter prospective observational registry.

## 2. Materials and Methods

### 2.1. Study Design and Population

This multicenter prospective observational study used data from the Korean Cardiac Arrest Research Consortium (KoCARC) [21]. The institutional review board of each center within the KoCARC approved the study protocol. Data were obtained from the KoCARC registry database in Korea for cardiac arrest events that occurred between 1 October 2015 and 30 June 2018. We included patients with OHCA transported to the emergency department (ED) by emergency medical service (EMS) with resuscitation [21]. We excluded OHCA patients with pre-hospital ROSC, terminal illness documented by medical records, patients under hospice care, pregnant patients, and patients with a previously documented ‘Do Not Resuscitate’ card [21]. We also excluded OHCA patients with specific nonmedical etiology such as trauma, drowning, poisoning, burn, asphyxia, or hanging [21].

### 2.2. OHCA Registry and Definition

The KoCARC is a hospital-based collaborative research network aiming to enhance the effectiveness and professionalism of research on the chain of survival. This registry was organized in 2014 after recruiting hospitals willing to participate voluntarily in the consortium [21]. Data were entered into a web-based electronic database registry using a standardized registry form [21]. Each participating hospital has a designated research coordinator who is responsible for ensuring data accuracy [21]. To ensure patient privacy, patient identifiers are anonymized [21]. This study used 21 independent and 1 dependent variables described below from the KoCARC registry: demographics including sex and age, CPR-related characteristics such as the presence of a witness or bystander CPR, initial electrocardiography (ECG) rhythms obtained pre-hospital arrival or in the ED, and provision of CPR by EMS or in the ED, CPR-related time variables such as basic life support interval (time from collapse to the initiation of chest compression attempts at the scene), defibrillation interval (time from collapse to the initiation of electrical shock), and pre-hospital interval (time from collapse to ED arrival), and clinical outcomes, including the presence of ROSC, ED outcomes (admitted, died, or transferred), hospital outcomes (discharged alive, died, or transferred), and neurological outcomes at the time of discharge. We did not use information such as laboratory test results or post-cardiac arrest treatment that was not available at the time of initial resuscitation.

The onset of cardiac arrest for a witnessed arrest was defined as the first recognition of unresponsiveness and apnea by anyone, including the first responder, lay-rescuer, or EMS. For unwitnessed arrests, it was defined as the time of recognition by the EMS. Sustained ROSC was defined as the restoration of a palpable pulse for at least 20 min. We defined downtime as the time from the onset of cardiac arrest to sustained ROSC. Survival to discharge was defined as discharge to home or transfer to another facility after admission to the hospital. Neurological outcome was quantified based on cerebral performance category (CPC) scores at the time of hospital discharge as follows: (1) no significant impairment, (2) moderate impairment but can complete activities of daily living, (3) severe impairment but conscious, (4) vegetative state or coma, and (5) death [22]. The primary endpoint was a good neurological outcome, defined as CPC scores of 1 or 2, while CPC scores of 3 to 5 were considered a poor outcome.

### 2.3. Methods (Machine Learning Algorithms)

This study developed models to predict outcomes using four supervised ML algorithms (regularized logistic regression (RLR), random forest (RF), XGBoost (XGB), and the voting classifier (VC) that was created with the three other models by a 1:1:1 ratio of votes). Details of the four machine learning methods applied in this study are described in the summary of used machine learning algorithm section of the Supplementary Material. In our dataset, the incidence of good neurological outcomes was highly imbalanced (Table 1 and Table S1). We performed data resampling in the two outcome groups [23] and tried to convert binary classification to multiclass classification by dividing the major or minority groups into two or more subgroups. However, these steps did not improve the metrics to be described. Stratified five-fold cross-validation was implemented to identify and validate the best-performing of the four ML models under generalized circumstances. The parameters were optimized by a grid-search algorithm for the highest Cohen’s kappa. The four best-performing trained ML models were evaluated based on the ratio of predicted good neurological outcome, area under the curve (AUC), log loss, and Brier score (BS) [24]. We also compared models using evaluation metrics such as sensitivity, specificity, positive predictive value (PPV), negative predictive value (NPV), F1-score, Cohen’s kappa, and net reclassification index (NRI). All scores of the four ML models and their 95% confidence intervals (CIs) were computed using 50 different samples from combinations of 10 imputation datasets (explained in Section 2.4 below) and five-fold cross-validation. Finally, local interpretable model-agnostic explanations of the VC model were performed. The open-source language, Python 3.7.6 [25], and its extension packages, scipy 1.2.1 [26], scikit-learn 0.22.2 [27], numpy 1.17.2 [28], xgboost 1.1.0 [29], matplotlib 3.0.2 [30], lime 0.1.1.33 [31], seaborn 0.10.0 [32], and venn 0.1.3 [33], were used in the ML.

### 2.4. Statistical Analysis

Continuous variables were expressed as medians with interquartile range (IQR). Categorical variables were reported as numbers and percentages. Mann–Whitney U tests were used to compare the values of continuous variables. Chi-square or Fisher’s exact tests were used for categorical variables. The variables in our data had missing values. By considering the missing values to be missing at random (MAR), it is possible to use multiple imputation by chained equations (MICE) to fill in the missing values [34,35,36]. All variables with missing values were sequentially imputed by a regression model created from the conditional marginal distributions of the other variables. This process was repeated until the missing values were no longer updated. The process that previously filled missing values of variables to estimate missing values of the other variables is called the chained equation, which is easily applicable to realistic problems. Ten imputed datasets were produced, taking into account the missing rate in variables and computing resources. For all reports, a two-sided *p* < 0.05 was considered to indicate a statistically significant difference. Statistical analyses were performed using R 3.6.1 (R Foundation for Statistical Computing, Vienna, Austria) and the mice 3.11.0 package [35].

## 3. Results

### 3.1. Baseline Statistics

We included a total of 5739 OHCA patients from the KoCARC registry database who met the selection criteria. After excluding 513 patients with pre-hospital ROSC, 105 patients (1.83%) had good neurological outcomes. In Table 1, the baseline characteristics are presented by classifying patients according to good or poor neurological outcomes. The large difference in the numbers of samples between the two groups indicated that our dataset was imbalanced. All variables except sex, hypertension, dyslipidemia, CPR and AED by a bystander, CPR by a machine, and endotracheal tube intubation differed between the two groups. Values were missing for 14 of the 21 variables (Figure S1). A total of 2149 patients had more than one missing value for the 21 predictors. The imputation results can be checked by comparing the observed and imputed data on the density plots shown in Figure S2.

### 3.2. Model Performances and Validation

**Figure 1:**
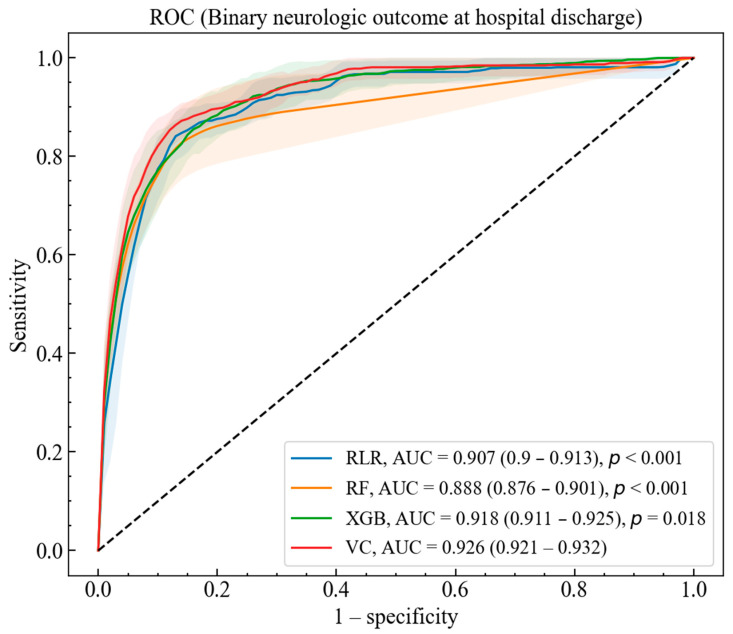
Receiver Operating Characteristic (ROC) and area under the curves (AUC) of regularized logistic regression (RLR), random forest (RF), extreme gradient boosting (XGB), and voting classifier (VC) for binary neurological outcome at hospital discharge. The colors of the curves and shaded regions represent the mean ROC and standard deviation of each model, respectively. The AUCs of the models and their 95% confidence intervals (CIs) are shown in the legend. The *p*-values for the hypothesis tests of the differences between the VC and the other models are also indicated in the legend.

The AUC (95% CI) of the RLR, LF, XGB, and VC models for predicting neurological outcomes among patients admitted to the ED for OHCA were 0.907 (0.900–0.913), 0.888 (0.876–0.901), 0.918 (0.911–0.925), and 0.926 (0.921–0.932), respectively. The receiver operating characteristics (ROC) curves and AUCs in Figure 1 are derived from the ML models by maximizing the Cohen’s kappa metric suitable for an imbalanced dataset. The performance of the VC model was better than those of the RLR, RF, and XGB models in terms of AUC. In our analysis, the thresholds of the RLR, RF, XGB, and VC models determined by Youden’s Index were 0.566, 0.0438, 0.0860, and 0.242, respectively. The sensitivity, specificity, PPV, and NPV of the four models according to the thresholds are presented in Figure S3.

The probabilities that the RLR, RF, XGB, and VC models predicted good neurological outcomes were 13.5%, 11.4%, 21.3%, and 6.61%, respectively; however, only 1.83% of patients showed good neurological outcomes in the test set. The log loss of random classifiers depending on the prevalence of classes in this case was about 0.1. The performance of all models but the RLR model was superior to that of random classifiers based on the value of 0.1. The Brier skill score (BSS) with Brier score of reference (BSR) was applied to understand more clearly the performance of the models [24]:

(1) $$\mathrm{BSS}=1-\frac{\mathrm{BS}}{\mathrm{BSR}}$$

where BS indicates the Brier score. If Brier skill score is zero, the trained model is a no-skill model like a random classifier, but if it is close to 1, it is an excellent performance model, while the range of Brier skill score is from −∞ to 1. We set the BSR to 0.333, which is the mean BS when random classifiers predicted neurological outcomes of 5709 patients for 100 iterations. All models but the RLR model performed better than random classifiers because their BSSs were larger than 0. 

The sensitivity, specificity, PPV, NPV, F1-score, Cohen’s kappa, and NRI were calculated using the thresholds in Table 2. F1-score, the harmonic mean of the PPV and sensitivity, is an indicator of the clinical usefulness of a model. Cohen’s kappa can assess observation and prediction reliability for binary classification. The confusion matrix shown in Table S2 indicated why Cohen’s kappa was used as an optimizing metric. The NRI adds the accuracy of the classification of two models for each positive and negative case. The 95% CIs of the NRI in Table 2 show that no model outperformed the RLR model.

**Figure 2:**
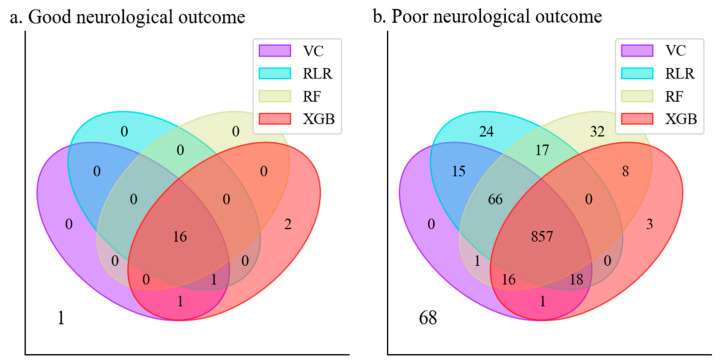
Venn diagrams of the predictions of neurological outcomes by model. (**a**) Comparison of predicted outcomes of the RLR, RF, XGB, and VC models for good neurological outcomes. (**b**) Comparison of predicted outcomes of the RLR, RF, XGB, and VC models for poor neurological outcomes.

The predictions of good and poor neurological outcomes for patients in a test set by the four models are shown in Figure 2. For good neurological outcomes, there was only one false-negative in which no model predicted good neurological outcomes. The XGB model predicted the most true-positives (20) and the least false-negatives (1). In contrast, the RLR model predicted the least true-positives (17) and the most false-negatives (4). Regarding poor neurological outcomes, no model predicted poor neurological outcomes for 68 patients in the test set. Both the RLR and RF models showed the best performance in that the number of unpredictable patients (129) was the smallest among the four ML models. Note that the results depended on the threshold for each model to predict the neurological outcomes. 

**Figure 3:**
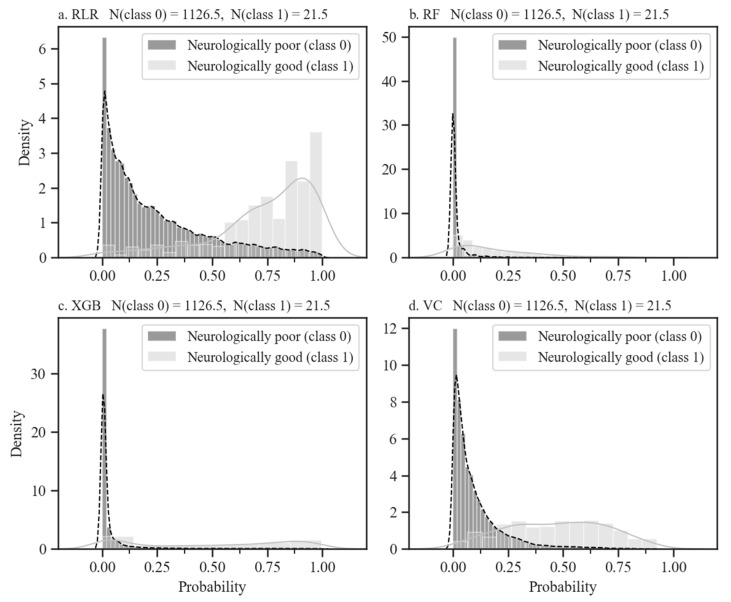
Probability histogram and kernel density estimation of neurological outcomes for four ML models. The title of the panels is the ML model name and the average number of samples per class of test sets. The probability histograms and kernel density estimations for good neurological outcomes are shown in silver bars and solid lines respectively, while those for poor neurological outcomes are represented as black bars and dashed lines, respectively. The panels of (**a**–**d**) are the probability distributions of the test sets by RLR, RF, XGB, and VC models, respectively.

The predicted probability density curves for the four models can be seen in Figure 3. On the *x*-axis, being closer to 1 indicates a good neurological outcome, while being closer to 0 indicates the opposite. The value of the *y*-axis is the density of samples in the test set with the same predicted probability, and the farther the distance between the two peaks, the better the model classifies the samples in the test set. Both the RF and XGB models’ thresholds are very sensitive, so classes can easily change with a small shift in them. However, the other two model classifications are robust to slight changes in thresholds. In particular, an overlapping range of two classes for the VC model is the narrowest. 

**Figure 4:**
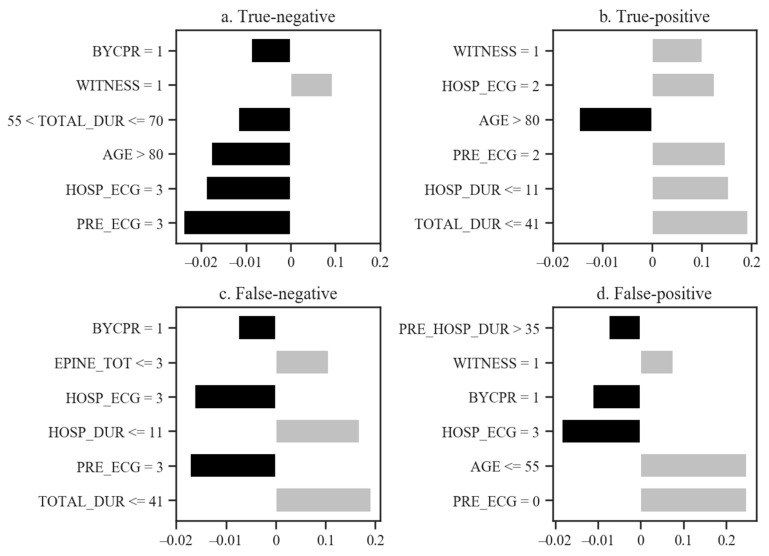
Why the model predicts the neurological outcomes of individual patients. Explainability of predicting neurological outcomes of (**a**) true-negative, (**b**) true-positive, (**c**) false-negative, and (**d**) false-positive cases. The *x*-axis is the probability of predicting prognosis for an individual patient. While other variables are fixed, a value of the *x*-axis for a feature speaks to how much to increase or decrease the predicted probability of the VC model. This VC model was trained using all variables in Table 1. The official names of the abbreviated variable names in this figure can be found in Appendix A, Table A1.

With true-negative, true-positive, false-negative, and false-positive cases, the reason the VC model, with the best AUC, predicted the neurological outcomes is explained in Figure 4. In true-negative cases, first pre-hospital ECG rhythm (PRE_ECG), first hospital ECG rhythm (HOSP_ECG), age (AGE), total duration of resuscitation (TOTAL_DUR), and pre-hospital bystander CPR (BYCPR) supported the poor neurological outcome (see Table S3 for an explanation of the variable names). Despite features like AGE, factors including TOTAL_DUR, HOSP_DUR, PRE_ECG, HOSP_ECG, and WITNESS contributed to good neurological outcomes in true-positive cases. In false-negative cases, PRE_ECG, HOSP_ECG, and BYCPR explained the poor neurological outcome; however, owing to TOTAL_DUR, duration of hospital resuscitation (HOSP_DUR), and total amount of epinephrine (EPINE_TOT), patients recovered without serious neurological damage. Lastly, the model predicted good neurological outcomes because of PRE_ECG, AGE, and WITNESS, while HOSP_ECG, BYCPR, and PRE_HOSP_ECG indicated poor neurological outcomes. Since the last two cases were near the boundary between the two classes, the top six variables supported the different results.

## 4. Discussion

In this study, we demonstrated the potential utility for ML models to predict good neurological outcomes of OHCA patients before ROSC. The VC model’s overall performance was better than those of the RLR, RF, and XGB models. Although current guidelines recommend that neurological outcomes in patients with OHCA be predicted based on multimodal approaches, in OHCA patients before ROSC who are just arriving at the ED, information to determine prognosis is insufficient [6]. It is challenging to quickly obtain the necessary information to determine the probability of achieving favorable neurological outcomes [7]. However, it is essential to develop strategies for predicting good neurological outcomes to tailor medical therapies for individual patients, including ECMO-CPR. While several studies have described predictive scores [8,9], to the best of our knowledge, this study is the first to apply ML methods in patients who have not achieved ROSC. 

Initial ECG rhythm, witnessed cardiac arrest, and age, which have good predictive power in other scores, are also seen as important indicators in our study [1,10,11]. Unlike other studies, our study used only information available from pre-hospital care at the time of arrival at the hospital. Nevertheless, our results showed a similar or better performance to other scores. Other studies have often included blood tests that can only be obtained at the hospital stage [1,10,37].

Johnsson et al. used a fully-connected one hidden layer structure to predict the prognosis of OHCA patients in the intensive care unit [37]. Owing to their balanced data, they applied an artificial neural network algorithm; however, the algorithm selected one hidden layer as an optimized structure because of a sample number below 1000. They did not apply RF and gradient boosting algorithms, which perform well on tabular-type datasets. The authors explicitly showed the procedure for global feature importance analysis but did not perform feature importance at the level of individual patients. Al-Dury et al. mainly focused on identifying the key predictors associated with 30-day survival using an RF algorithm [38]. An elaborate permutation that can handle the scale of the variables and the correlation between predictors measured the feature importance of the RF model. To clarify the association between 30-day survival and key features such as age, time to CPR, and time to EMS arrival, the authors used partial dependence plots by marginalizing over the other features. While they described in detail the global correlations between response and predictors, the feature importance at the individual patient level for false-positive or false-negative cases was not shown. Kwon et al. developed high-performance ML models based on several algorithms [20]. The accuracy of the prediction of each model and the stability of the predictions between models were sufficiently analyzed using various scores. Although global feature importance was performed, they did not assess the explainability for predicting a patient’s prognosis. Seki et al. also showed excellent performance using a ML-based model to predict 1-year survival of OHCA with presumed cardiac etiology. While their goal differed slightly from that in our study, they also demonstrated the potential of ML-based approaches. Our study has the following strengths. The KoCARC is a well-defined hospital-based collaborative research network [21]. There were strict predefined protocol-based criteria for inclusion and treatment and rules for neurological prognostication. Also, since our study only targeted patients who had not achieved ROSC at ED arrival, it has practical and clinical value. Finally, we assessed the importance of the variables used in this study in various ways to provide explanatory power in future research. It will help avoid inappropriate withdrawal of life-sustaining treatment in patients who may otherwise achieve meaningful neurological recovery. Further prospective validation study will be needed to confirm our result.

Our study has several limitations. Although the dataset was a nationwide multicenter registry, there may be selection bias. Also, since the data are of Asian patients, further research is needed to generalize to other races. According to the recent systematic review literature, the survival discharge rate is around 8.8% [39]. In our study, the rate of survival to hospital discharge was lower than that result (5.1% vs. 8.8%). Also, the proportion of OHCA patients without prehospital ROSC is higher than that of other studies. Both aspects may bias the results of this study, so care should be taken when interpreting them. Values were missing for 14 of the 21 variables (Figure S1). To minimize the effect of these missing data, we used MICE to fill in the missing values [34,35,36]. All variables with missing values were sequentially imputed by a regression model created from the conditional marginal distributions of the other variables. Missing values are known to significantly impact the prognostic models in medical fields [33] and applying the MICE to impute missing values might be burdensome in the real clinical practice. Therefore, we trained the models in three other convenient ways: median imputation, no imputation, and the complete dataset dropped the variables with missing values. Interestingly, there was little performance degradation of the models in our study by the first two methods (Figures S4–S6). Lastly, the low PPV and Cohen’s kappa of our models would be that the ratio of cases for the good neurological outcome in our dataset was very low, and 4 models could be trained to produce many false-positive cases.

## 5. Conclusions

We demonstrated the potential utility of a ML model to predict good neurological outcomes of OHCA patients before ROSC. The VC model’s overall performance was better than those of the RLR, RF, and XGB models. Our study used data from a well-defined multicenter registry for neurological prognostication. Since our study only targeted patients without ROSC at ED arrival, it has more practical and clinical value.

## Figures and Tables

**Table 1:** Basic statistics of the variables of the study subjects categorized according to neurological outcome.

Predictor Variables		Good Neurological Outcomes (*n* = 105)	Poor Neurological Outcomes (*n* = 5634)	*p*-Value
Demographics	Age (median, (IQR))	57 (47–66)	71 (58–80)	<0.001
	Male sex (*n*, %)	72 (68.6%)	3596 (63.8%)	0.367
	Hypertension (*n*, %)	45 (43.0%)	2268 (40.3%)	0.743
	Diabetes mellitus (*n*, %)	22 (21.0%)	1438 (25.5%)	0.099
	Dyslipidemia (*n*, %)	5 (4.76%)	259 (4.60%)	0.971
Pre-hospital	Witnessed (*n*, %)	85 (81.0%)	3149 (55.9%)	<0.001
	Occurrence at house (*n*, %)	39 (37.1%)	3687 (65.4%)	<0.001
	Bystander CPR (*n*, %)	39 (37.1%)	2702 (48.0%)	0.051
	Automated external defibrillation use (*n*, %)	3 (2.85%)	55 (0.976%)	0.152
	First ECG rhythm (*n*, %)			<0.001
	Ventricular fibrillation	47 (44.8%)	648 (11.5%)	
	Pulseless ventricular tachycardia	2 (1.90%)	26 (0.461%)	
	Pulseless electrical activity	31 (29.5%)	1150 (20.4%)	
	Asystole	12 (11.4%)	3451 (61.3%)	
	Airway (*n*, %)	72 (68.6%)	1083 (19.2%)	<0.001
			4483 (79.6%)	0.007
Hospital	Endotracheal intubation (*n*, %)	99 (94.3%)	5015 (89.0%)	0.347
	First ECG rhythm (*n*, %)			<0.001
	Ventricular fibrillation	37 (35.2%)	290 (5.14%)	
	Pulseless ventricular tachycardia	1 (0.952%)	13 (0.231%)	
	Pulseless electrical activity	38 (36.2%)	1078 (19.1%)	
	Asystole	24 (22.9%)	4114 (73.0%)	
	Use of mechanical compressor (*n*, %)	17 (16.2%)	925 (16.4%)	0.955
	Total epinephrine (mg, median, (IQR))	2 (1–4)	6 (3–9)	<0.001
	Defibrillation number (median, (IQR))	0 (0–3)	0 (0–0)	<0.001
Duration	Duration of resuscitation, (min, median, (IQR))			
	Total	27 (15–43)	55 (42–71)	<0.001
	Pre-hospital	17 (7–26)	26 (19–36)	<0.001
	Hospital	6 (3–12)	20 (11–30)	<0.001
	No flow time, (min, median, (IQR))	0 (0–5)	0 (0–8)	0.016

CPR: Cardiopulmonary resuscitation, IQR: Interquartile range, ECG: electrocardiography.

**Table 2:** The ratio of predicted good neurological outcome, AUC, log loss, Brier score, and evaluation metrics for the models and their 95% CIs.

Model	Actual Survival	RLR	RF	XGB	VC
Predicted survival	0.019	0.226 (0.218–0.234)	0.156 (0.149–0.163)	0.155 (0.153–0.158)	0.0819 (0.0747–0.089)
AUC	n.a.	0.893 (0.883–0.903)	0.881 (0.869–0.892)	0.925 (0.919–0.931)	0.925 (0.917–0.933)
Brier score	n.a.	0.389 (0.381–0.397)	0.138 (0.124–0.151)	0.107 (0.102–0.113)	0.146 (0.143–0.149)
Log loss	n.a.	0.119 (0.116–0.121)	0.0153 (0.0149–0.0160)	0.0302 (0.0283–0.0320)	0.0318 (0.0308–0.0330)
Sensitivity	n.a.	0.857 (0.842–0.872)	0.827 (0.804–0.850)	0.836 (0.804–0.868)	0.857 (0.843–0.871)
Specificity	n.a.	0.786 (0.778–0.793)	0.857 (0.85–0.863)	0.851 (0.836–0.866)	0.865 (0.858–0.873)
PPV	n.a.	0.0702 (0.0679–0.072)	0.0983 (0.095–0.102)	0.104 (0.0954–0.113)	0.109 (0.104–0.114)
NPV	n.a.	0.997 (0.996–0.997)	0.996 (0.996–0.997)	0.997 (0.996–0.997)	0.997(0.997–0.997)
F1-score	n.a.	0.819 (0.811–0.826)	0.839 (0.828–0.849)	0.836 (0.823–0.848)	0.86 (0.854–0.866)
Kappa	n.a.	0.0991 (0.095–0.103)	0.147 (0.142–0.153)	0.155 (0.142–0.167)	0.165 (0.158–0.173)
NRI	n.a.	n.a.	0.0404 (0.0132–0.0680)	0.0448 (0.0215–0.0680)	0.0796 (0.0638–0.0960)

PPV: positive predictive value, NPV: negative predictive value, F1-score: harmonic mean of PPV and sensitivity, Kappa: Cohen’s kappa, agreement of two raters, NRI: net reclassification improvement, the quantification of the improvement in the reclassification performance of the new model, n.a.: not applicable.

## Data Availability

The data presented in this study can be available on request from the corresponding author. The data are not publicly available due to privacy.

## Supplementary Materials

The following are available online at https://www.mdpi.com/2077-0383/10/5/1089/s1, Summary of used machine learning algorithms; Table S1: Baseline statistics, Table S2: Mean confusion matrix of the four ML models for the test sets, Figure S1: Heatmap of the frequency of missing values, Figure S2: Comparison of the distributions for 10 imputed datasets (red line) and observed data (blue line) using the mice package in R, Figure S3: Sensitivity, specificity, positive predictive value, and negative predictive value as a function of the threshold for the four ML models. Figure S4: The AUROC of 4 ML models trained with dataset imputed by the median of variables containing missing values, Figure S5: The AUROC of the XGB for dataset with missing values without data imputation, Figure S6: The AUROC of the 4 ML models for the complete dataset with dropped variables containing missing values.

## Appendix A

**Table A1:** Convenient variable names corresponding to the official variable names.

Official Variable Names	Convenient Variable Names
Demographics, Age	AGE
Demographics, Male sex	SEX
Demographics, Hypertension	HTN
Demographics, Diabetes mellitus	DM
Demographics, Dyslipidemia	DYSLIPID
Pre-hospital, Witnessed	WITNESS
Pre-hospital, Occurrence at house	OCC_HOUSE
Pre-hospital, Bystander CPR	BYCPR
Pre-hospital, Automated external defibrillation use	BYAED
Pre-hospital, First ECG rhythm, ventricular fibrillation	PRE_ECG: 0
Pre-hospital, First ECG rhythm, pulseless ventricular tachycardia	PRE_ECG: 1
Pre-hospital, First ECG rhythm, pulseless electrical activity	PRE_ECG: 2
Pre-hospital, First ECG rhythm, asystole	PRE_ECG: 3
Pre-hospital, Defibrillation	PRE_DEFIB
Pre-hospital, Airway	PRE_AIRWAY
Hospital, Endotracheal intubation	ENDO_INTU
Hospital First ECG rhythm, Ventricular fibrillation	HOSP_ECG: 0
Hospital First ECG rhythm, Pulseless Ventricular Tachycardia	HOSP_ECG: 1
Hospital First ECG rhythm, Pulseless Electrical Activity	HOSP_ECG: 2
Hospital First ECG rhythm, Asystole	HOSP_ECG: 3
Hospital, Use of mechanical compressor	MECH_CPR
Hospital, Total epinephrine	EPINE_TOT
Hospital, Defibrillation number	DEFIB_N
Duration of resuscitation, Total	TOTAL_DUR
Duration of resuscitation, Pre-hospital	PRE_HOSP_DUR
Duration of resuscitation, Hospital	HOSP_DUR
Duration, No flow time	NO_FLOW_TIME

## References

[1] Shih H.-M., Chen Y.-C., Chen C.-Y., Huang F.-W., Chang S.-S., Yu S.-H., Wu S.-Y., Chen W.-K. (2019). Derivation and Validation of the SWAP Score for Very Early Prediction of Neurologic Outcome in Patients with Out-of-Hospital Cardiac Arrest. Ann. Emerg. Med..

[2] Sasson C., Rogers M.A.M., Dahl J., Kellermann A.L. (2010). Predictors of survival from out-of-hospital cardiac arrest: A systematic review and meta-analysis. Circ. Cardiovasc. Qual. Outcomes.

[3] Berdowski J., Berg R.A., Tijssen J.G.P., Koster R.W. (2010). Global incidences of out-of-hospital cardiac arrest and survival rates: Systematic review of 67 prospective studies. Resuscitation.

[4] Yoon J.C., Kim Y.J., Ahn S., Jin Y.H., Lee S.W., Song K.J., Shin S.D., Hwang S.O., Kim W.Y., Korean Cardiac Arrest Research Consortium KoCARC (2019). Factors for modifying the termination of resuscitation rule in out-of-hospital cardiac arrest. Am. Heart J..

[5] Morrison L.J., Visentin L.M., Kiss A., Theriault R., Eby D., Vermeulen M., Sherbino J., Verbeek P.R., TOR Investigators (2006). Validation of a rule for termination of resuscitation in out-of-hospital cardiac arrest. N. Engl. J. Med..

[6] Nolan J.P., Soar J., Cariou A., Cronberg T., Moulaert V.R.M., Deakin C.D., Bottiger B.W., Friberg H., Sunde K., Sandroni C. (2015). European Resuscitation Council and European Society of Intensive Care Medicine Guidelines for Post-resuscitation Care 2015: Section 5 of the European Resuscitation Council Guidelines for Resuscitation 2015. Resuscitation.

[7] Kim Y.J., Kim M.-J., Koo Y.S., Kim W.Y. (2020). Background Frequency Patterns in Standard Electroencephalography as an Early Prognostic Tool in Out-of-Hospital Cardiac Arrest Survivors Treated with Targeted Temperature Management. J. Clin. Med..

[8] Callaway C.W., Donnino M.W., Fink E.L., Geocadin R.G., Golan E., Kern K.B., Leary M., Meurer W.J., Peberdy M.A., Thompson T.M. (2015). Part 8: Post–Cardiac Arrest Care. Circulation.

[9] Rittenberger J.C., Tisherman S.A., Holm M.B., Guyette F.X., Callaway C.W. (2011). An early, novel illness severity score to predict outcome after cardiac arrest. Resuscitation.

[10] Adrie C., Cariou A., Mourvillier B., Laurent I., Dabbane H., Hantala F., Rhaoui A., Thuong M., Monchi M. (2006). Predicting survival with good neurological recovery at hospital admission after successful resuscitation of out-of-hospital cardiac arrest: The OHCA score. Eur. Heart J..

[11] Maupain C., Bougouin W., Lamhaut L., Deye N., Diehl J.-L., Geri G., Perier M.-C., Beganton F., Marijon E., Jouven X. (2016). The CAHP (Cardiac Arrest Hospital Prognosis) score: A tool for risk stratification after out-of-hospital cardiac arrest. Eur. Heart J..

[12] Esteva A., Kuprel B., Novoa R.A., Ko J., Swetter S.M., Blau H.M., Thrun S. (2017). Dermatologist-level classification of skin cancer with deep neural networks. Nature.

[13] Fauw J., Ledsam J.R., Romera-Paredes B., Nikolov S., Tomasev N., Blackwell S., Askham H., Glorot X., O’Donoghue B., Visentin D. (2018). Clinically applicable deep learning for diagnosis and referral in retinal disease. Nat. Med..

[14] Rajkomar A., Oren E., Chen K., Dai A.M., Hajaj N., Hardt M., Liu P.J., Liu X., Marcus J., Sun M. (2018). Scalable and accurate deep learning with electronic health records. NPJ Digit. Med..

[15] Zhang J., Gajjala S., Agrawal P., Tison G.H., Hallock L.A., Beussink-Nelson L., Lassen M.H., Fan E., Aras M.A., Jordan C. (2018). Fully Automated Echocardiogram Interpretation in Clinical Practice. Circulation.

[16] Douglas P.S., De Bruyne B., Pontone G., Patel M.R., Nørgaard B.L., Byrne R.A., Curzen N., Purcell I., Gutberlet M., Rioufol G. (2016). 1-Year Outcomes of FFRCT-Guided Care in Patients With Suspected Coronary Disease: The PLATFORM Study. J. Am. Coll. Cardiol..

[17] Shung D., Simonov M., Gentry M., Au B., Laine L. (2019). Machine Learning to Predict Outcomes in Patients with Acute Gastrointestinal Bleeding: A Systematic Review. Dig. Dis. Sci..

[18] Liu R., Greenstein J.L., Granite S.J., Fackler J.C., Bembea M.M., Sarma S.V., Winslow R.L. (2019). Data-driven discovery of a novel sepsis pre-shock state predicts impending septic shock in the ICU. Sci. Rep..

[19] Wilson F.P., Shashaty M., Testani J., Aqeel I., Borovskiy Y., Ellenberg S.S., Feldman H.I., Fernandez H., Gitelman Y., Lin J. (2015). Automated, electronic alerts for acute kidney injury: A single-blind, parallel-group, randomised controlled trial. Lancet.

[20] Kwon J.-M., Jeon K.-H., Kim H.M., Kim M.J., Lim S., Kim K.H., Song P.S., Park J., Choi R.K., Oh B.-H. (2019). Deep-learning-based out-of-hospital cardiac arrest prognostic system to predict clinical outcomes. Resuscitation.

[21] Kim J.Y., Hwang S.O., Shin S.D., Yang H.J., Chung S.P., Lee S.W., Song K.J., Hwang S.S., Cho G.C., Moon S.W. (2018). Korean Cardiac Arrest Research Consortium (KoCARC): Rationale, development, and implementation. Clin. Exp. Emerg. Med..

[22] Jennett B., Bond M. (1975). Assessment of outcome after severe brain damage. Lancet.

[23] Tomek I. (1976). Two modications of CNN. Syst. Man Cypernetics IEEE Trans..

[24] Stefanova L., Krishnamurti T.N. (2002). Interpretation of Seasonal Climate Forecast Using Brier Skill Score, The Florida State University Superensemble, and the AMIP-I Dataset. J. Clim..

[25] Van Rossum G., Drake F.L. (1995). Python Tutorial.

[26] Virtanen P., Gommers R., Oliphant T.E., Haberland M., Reddy T., Cournapeau D., Burovski E., Peterson P., Weckesser W., Bright J. (2020). SciPy 1.0: Fundamental Algorithms for Scientific Computing in Python. Nat. Methods.

[27] Pedregosa F., Varoquaux G., Gramfort A., Michel V., Thirion B., Grisel O., Blondel M., Prettenhofer P., Weiss R., Dubourg V. (2011). Scikit-learn: Machine Learning in Python. J. Mach. Learn. Res..

[28] Oliphant T.E. (2006). A Guide to NumPy.

[29] Chen T., Guestrin C. (2016). XGBoost: A Scalable Tree Boosting System. Proceedings of the 22nd ACM SIGKDD International Conference on Knowledge Discovery and Data Mining.

[30] Hunter J.D. (2007). Matplotlib: A 2D graphics environment. Comput. Sci. Eng..

[31] Ribeiro M.T., Singh S. Why should i trust you? Explaining the predictions of any classifier. Proceedings of the 22nd ACM SIGKDD International Conference on Knowledge Discovery and Data Mining.

[32] mwaskom/seaborn: v0.8.1 (September 2017).

[33] Sharafoddini A., Dubin J.A., Maslove D.M., Lee J. (2019). A New Insight Into Missing Data in Intensive Care Unit Patient Profiles: Observational Study. JMIR Med. Inform..

[34] Azur M.J., Stuart E.A., Frangakis C., Leaf P.J. (2011). Multiple imputation by chained equations: What is it and how does it work?. Int. J. Methods Psychiatr. Res..

[35] Van Buuren S., Groothuis-Oudshoorn K. (2011). mice: Multivariate Imputation by Chained Equations in R. J. Stat. Softw..

[36] Papageorgiou G., Grant S.W., Takkenberg J.J.M., Mokhles M.M. (2018). Statistical primer: How to deal with missing data in scientific research?. Interact. Cardio Vasc. Thorac. Surg..

[37] Johnsson J., Björnsson O., Andersson P., Jakobsson A., Cronberg T., Lilja G., Friberg H., Hassager C., Kjaergard J., Wise M. (2020). Artificial neural networks improve early outcome prediction and risk classification in out-of-hospital cardiac arrest patients admitted to intensive care. Critical Care.

[38] Al-Dury N., Ravn-Fischer A., Hollenberg J., Israelsson J., Nordberg P., Strömsöe A., Axelsson C., Herlitz J., Rawshani A. (2020). Identifying the relative importance of predictors of survival in out of hospital cardiac arrest: A machine learning study. Scand. J. Trauma Resusc. Emerg. Med..

[39] Yan S., Gan Y., Jiang N., Wang R., Chen Y., Luo Z., Zong Q., Chen S., Lv C. (2020). The global survival rate among adult out-of-hospital cardiac arrest patients who received cardiopulmonary resuscitation: A systematic review and meta-analysis. Critical Care.

