# Supplementary material for: Prediction of Neurologically Intact Survival in Cardiac Arrest Patients without Pre-Hospital Return of Spontaneous Circulation: Machine Learning Approach
Source: J Clin Med. 2021 Mar 5;10(5):1089. doi: 10.3390/jcm10051089 (PMC7961400; doi:10.3390/jcm10051089)
Supplement: Supplementary file 1 [file jcm-10-01089-s001.pdf]

Summary of used machine learning algorithms

Logistic regression is a basic statistical algorithm to predict the possibility of an event with a combination of predicting variables [1]. In particular, regularized logistic regression (RLR) prevents the model from learning peculiarity or noise from training datasets and helps to better predict data that it has not seen [2]. In other words, by inserting bias, the variance is lowered to improve the model's general performance. Random forest (RF) makes many decision trees and predicts the outcome by majority voting and the mean value of each decision tree result [3]. The input of the decision tree is a subset of randomly selected features, which are partitioned in the direction of decreasing entropy to form the decision tree. This approach leads to easy overfitting of the models; thus, RF combined with the results of many decision trees generally perform well to predict outcomes. Unlike RF, XGBoost (XGB) makes a tree that assigns more weight to higher errors from previous decision trees but is similar to RF in that it is an ensemble learning algorithm where the outcome of sequentially built trees are combined to predict the final outcome [4]. XGB can quickly converge to the minimum of the loss function by computing the second-order gradient of the loss function. In addition, it can handle missing values in variables and may prevent models from overfitting by regularizing (L1 and L2). Finally, stable outcomes were predicted by a voting classifier (VC) model that combined the other three models created by each ML algorithm [5]. The VC model blended the RLR, RF, and XGB at a 1:1:1 ratio, respectively.

1. Cox DR. The regression analysis of binary sequence. Journal of the Royal Statistical Society. 1958;20:28.

2. Salehi F, Abbasi E, Hassibi B. The Impact of Regularization on High-dimensional Logistic Regression. 2019;:12005–15.

3. Breiman L. Random forests. Machine learning. 2001;45:28.

4. Chen T, Guestrin C. XGBoost: A Scalable Tree Boosting System. Proceedings of the 22nd ACM SIGKDD International Conference on Knowledge Discovery and Data Mining. Association for Computing Machinery; 2016. pp. 785–94.

5. Opitz D, Maclin R. Popular Ensemble Methods: An Empirical Study. Journal of Artificial Intelligence Research. 1999;11:30.

Table S1. Baseline statistics

Predictor variables		n = 5739
Demographics	Age (median, [IQR])	71 [58 - 80]
	Male sex (n, %)	3668 (63.9%)
	Hypertension (n, %)	2313 (40.3%)
	Diabetes mellitus (n, %)	1460 (25.4%)
	Dyslipidemia (n, %)	264 (4.60%)
Pre-hospital	Witnessed (n, %)	3234 (56.4%)
	Occurrence at house (n, %)	3726 (64.9%)
	Bystander CPR (n, %)	2741 (47.8%)
	Automated external defibrillation use (n, %)	58 (1.01%)
	First ECG rhythm (n, %)	
	Ventricular fibrillation	695 (12.1%)
	Pulseless ventricular tachycardia	28 (0.487%)
	Pulseless electrical activity	1181 (20.6%)
	Asystole	3463 (60.3%)
	Defibrillation (n, %)	1136 (19.8%)
	Airway (n, %)	4555 (79.4%)
Hospital	Endotracheal intubation (n, %)	5114 (89.1%)
	First ECG rhythm (n, %)	
	Ventricular fibrillation	327 (5.70%)
	Pulseless ventricular tachycardia	14 (0.243%)
	Pulseless electrical activity	1116 (19.5%)
	Asystole	4138 (72.1%)
	Use of mechanical compressor (n, %)	942 (16.4%)
	Total epinephrine (mg, median, [IQR])	6 [3 - 9]
	Defibrillation number (median, [IQR])	0 [0 - 0]
Duration	Duration of resuscitation, (min, median, [IQR])	
	Total	55 [41 - 71]
	Pre-hospital	26 [19 - 35]
	Hospital	20 [10 - 30]
	No flow time (min, median, [IQR])	0 [0 - 8]

Table S2. Mean confusion matrix of the four machine learning (ML) models for the test sets

RLR neurological outcome (threshold = 0.566)			
RLR	N (Prediction)	P (Prediction)	Total
N (Actual)	988.64	138.16	1126.8
P (Actual)	3.78	17.22	21
Total	992.42	155.38	1147.8
RF neurological outcome (threshold = 0.0438)			
RF	N (Prediction)	P (Prediction)	Total
N (Actual)	1012.08	114.72	1126.8
P (Actual)	4.74	16.26	21
Total	1016.82	130.98	1147.8
XGB neurological outcome (threshold = 0.0860)			
XGB	N (Prediction)	P (Prediction)	Total
N (Actual)	1005.18	121.62	1126.8
P (Actual)	5.28	15.72	21
Total	1010.46	137.34	1147.8
VC neurological outcome (threshold = 0.242)			
VC	N (Prediction)	P (Prediction)	Total
N (Actual)	1006.26	120.54	1126.8
P (Actual)	3.88	17.12	21
Total	1010.14	137.66	1147.8

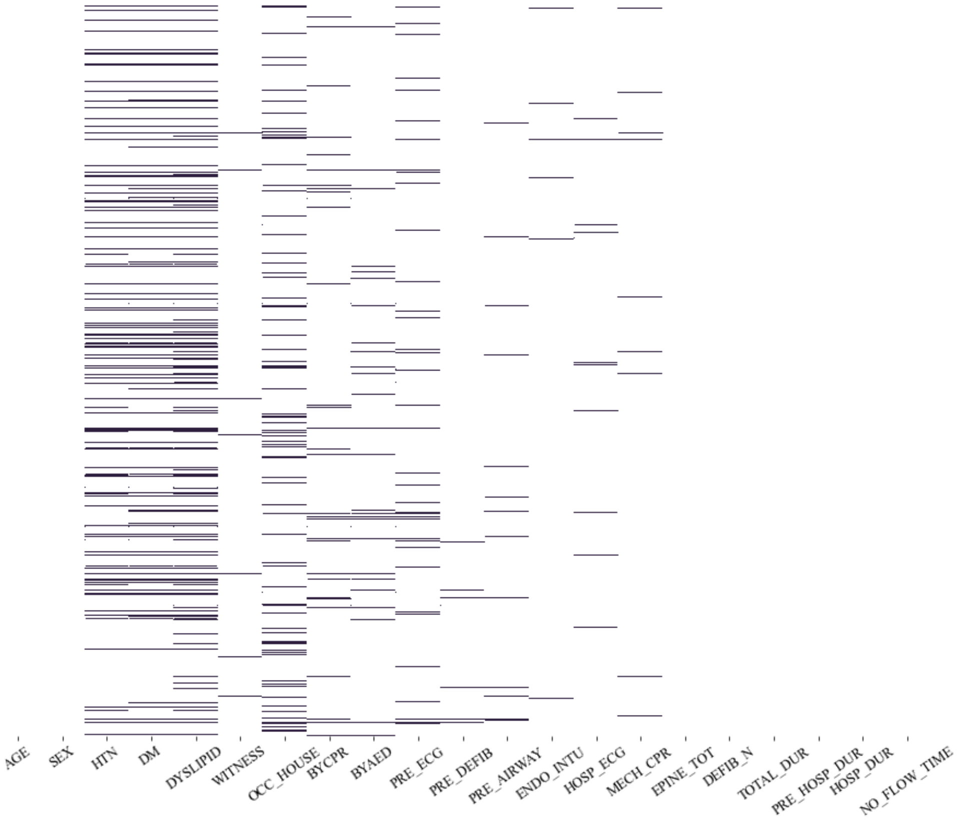

Figure S1. Heatmap of the frequency of missing values. The black lines indicate missing values in the predictor variables. The corresponding official variable names of the convenient variable names shown in this figure are indicated in Table S3, Additional File 1.

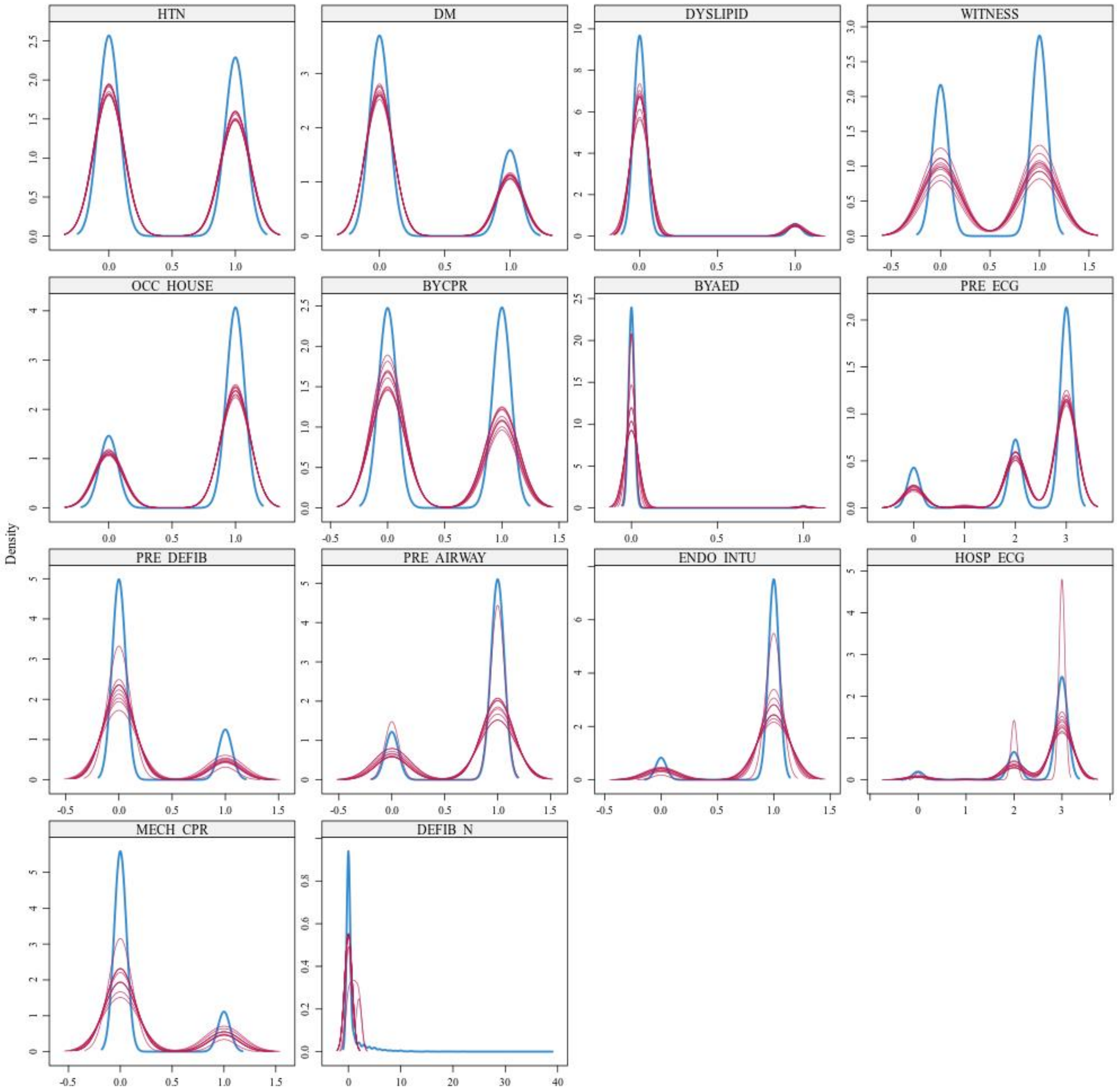

Figure S2. Comparison of the distributions for 10 imputed datasets (red line) and observed data (blue line) using the *mice* R package. The corresponding official variable names for the convenient variable names shown in this figure are indicated in Table S3, Additional File 1.

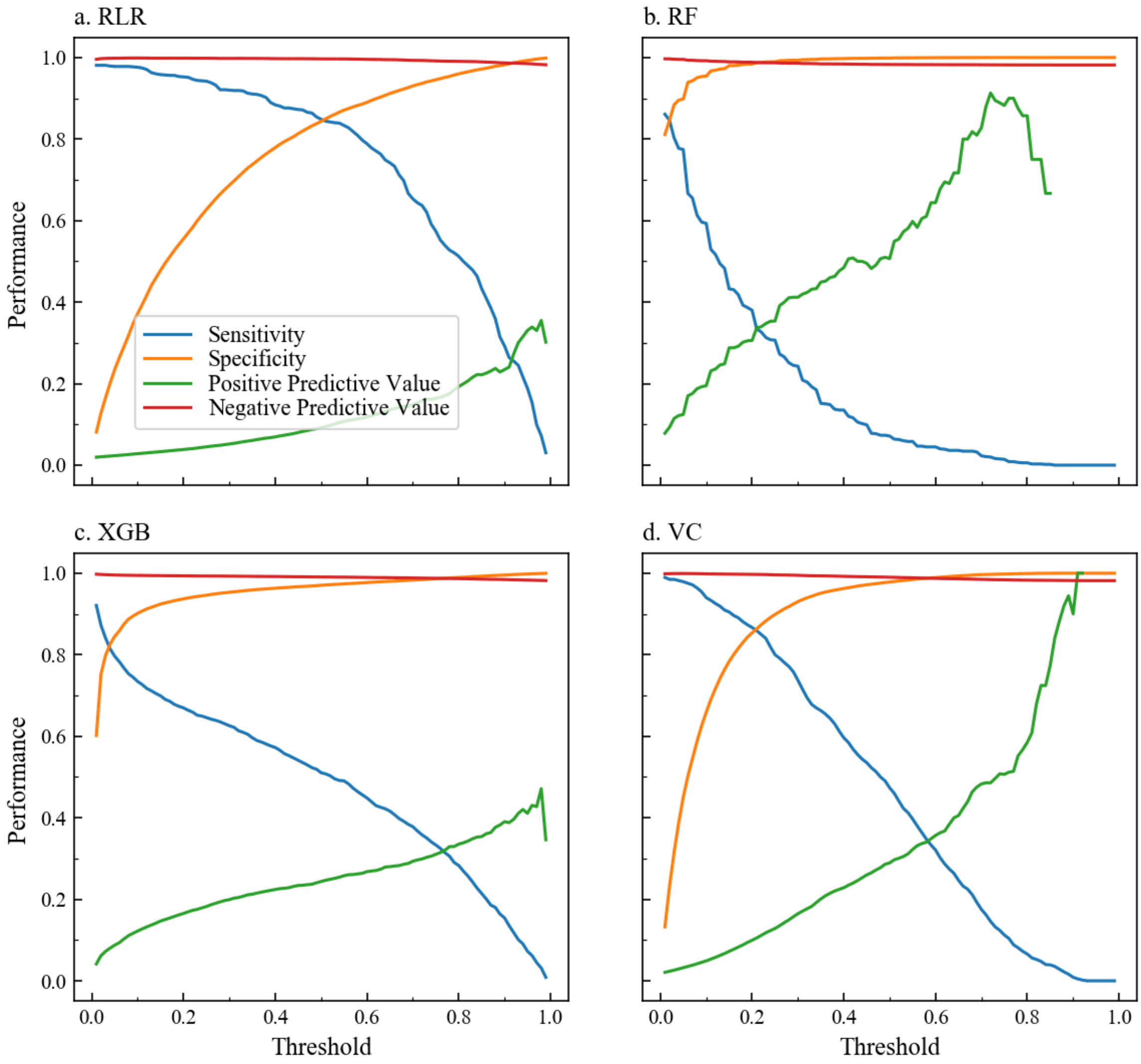

Figure S3. Sensitivity, specificity, positive predictive value, and negative predictive value as a function of the thresholds for the four ML models

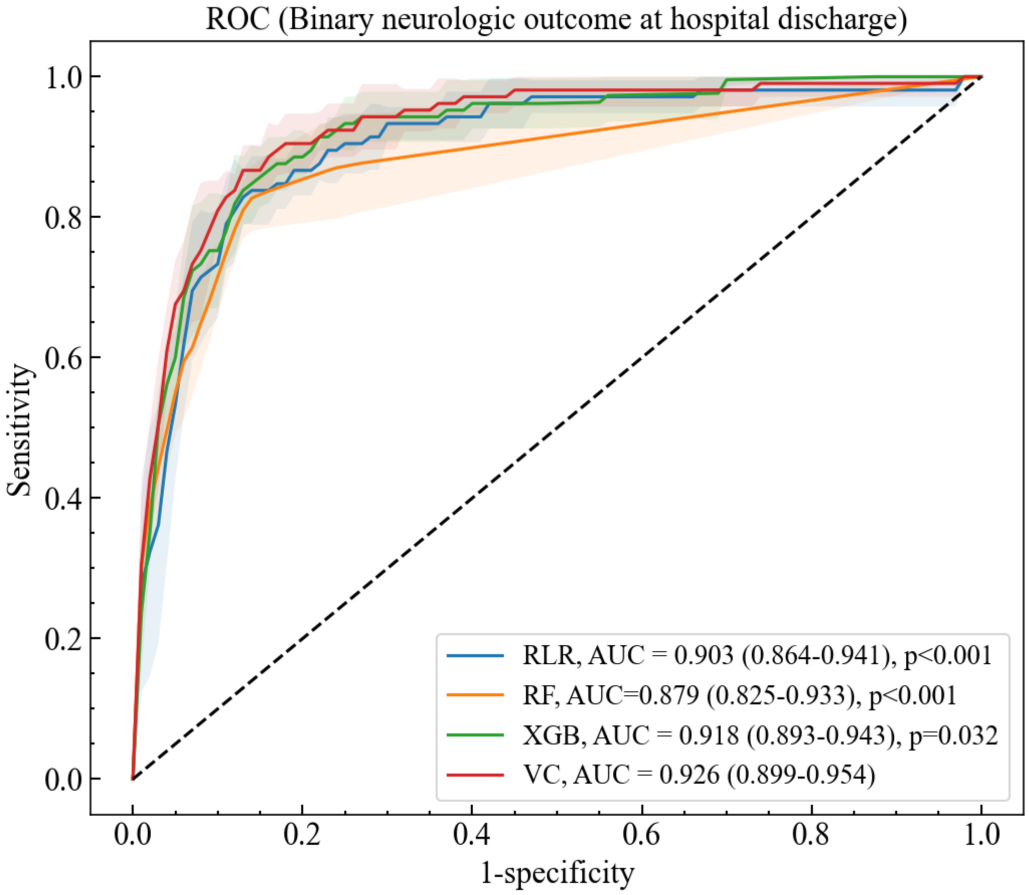

Figure 4S. The AUROC of 4 ML models trained with data-set imputed by the median of variables containing missing values

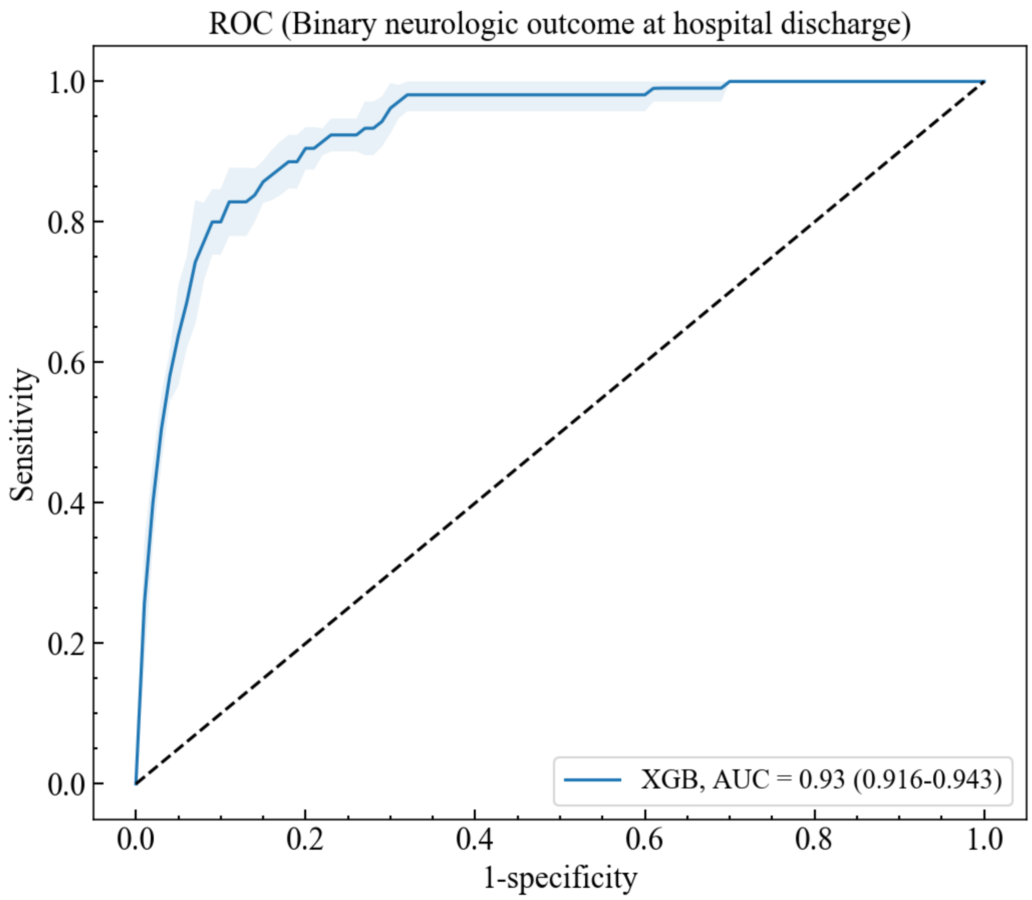

Figure 5S. The AUROC of the XGB for data-set with missing values without data imputation

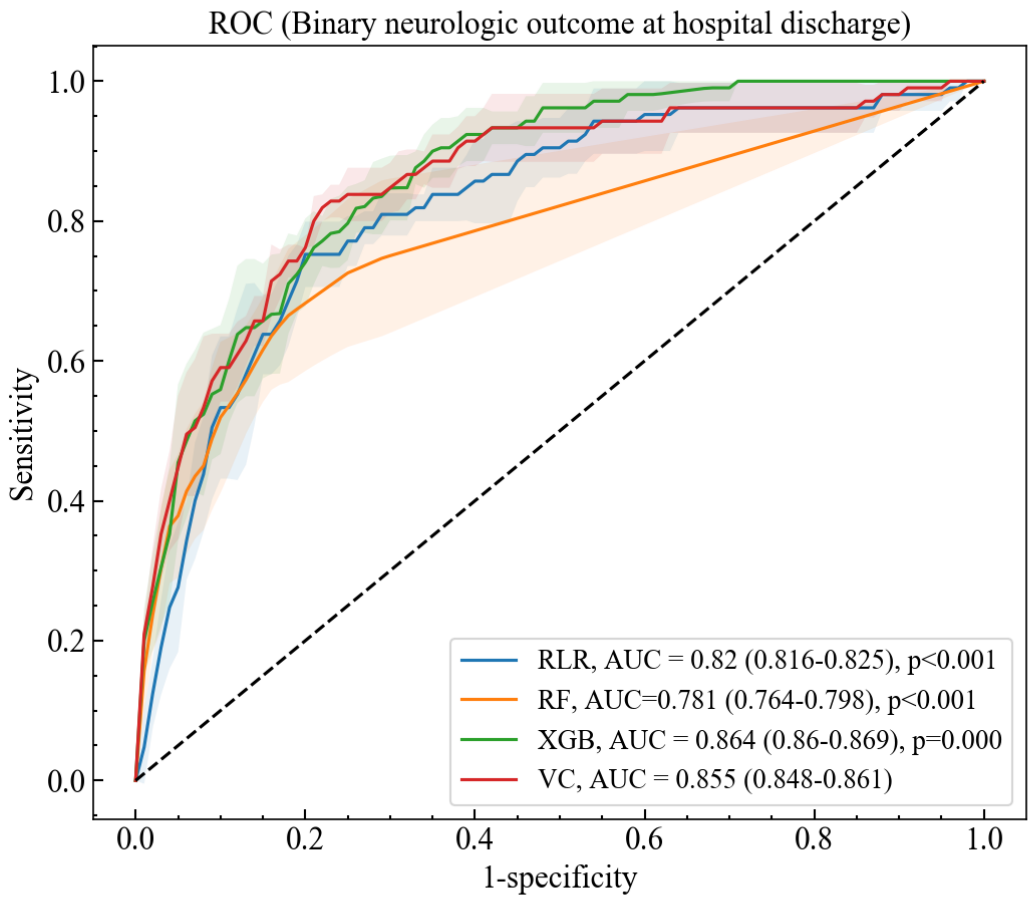

Figure 6S. The AUROC of 4 ML models for *the complete* data-set with dropped variables containing missing values
